# Dysregulated expression of slingshot protein phosphatase 1 (SSH1) disrupts circadian rhythm and WNT signaling associated to hepatocellular carcinoma pathogenesis

**DOI:** 10.18632/aging.205064

**Published:** 2023-10-13

**Authors:** Shiue-Wei Lai, Yi-Chiao Cheng, Wen-Chien Huang, Vijesh Kumar Yadav, Iat-Hang Fong, Chi-Tai Yeh, Ching-Kuo Yang, Wei-Hwa Lee, Ming-Yao Chen

**Affiliations:** 1 Division of Hematology/Oncology, Department of Internal Medicine, Tri-Service General Hospital, National Defense Medical Center, Taipei City 114, Taiwan; 2 Division of Colon and Rectal Surgery, Department of Surgery, Tri-Service General Hospital, National Defense Medical Center, Taipei City 114, Taiwan; 3 Division of Thoracic Surgery, Department of Surgery, MacKay Memorial Hospital, Taipei 104, Taiwan; 4 Department of Medicine, MacKay Medical College, New Taipei City 252, Taiwan; 5 Division of Gastroenterology and Hepatology, Department of Internal Medicine, School of Medicine, College of Medicine, Taipei Medical University, Taipei City 110, Taiwan; 6 Division of Gastroenterology and Hepatology, Department of Internal Medicine, Shuang Ho Hospital, New Taipei City 235, Taiwan; 7 Continuing Education Program of Food Biotechnology Applications, College of Science and Engineering, National Taitung University, Taitung 95092, Taiwan; 8 Division of Colorectal Surgery, Department of Surgery, Mackay Memorial Hospital, Taipei City 110, Taiwan; 9 Department of Pathology, Taipei Medical University-Shuang Ho Hospital, New Taipei City 235, Taiwan; 10 TMU Research Center for Digestive Medicine, Taipei Medical University, Taipei 110, Taiwan

**Keywords:** hepatocellular carcinoma, SSH1, circadian rhythm, WNT/β-catenin signaling, sennoside A therapy

## Abstract

Growing evidence underscores the circadian rhythm’s essential function in liver stability and disease. Its disruption is progressively linked with metabolic issues, oncogene triggers, and heightened cancer susceptibility. Research points to slingshot protein phosphatase 1 (SSH1), a modulator of cofilin-1 (CFL-1), as instrumental in the reformation of the actin cytoskeleton, thereby impacting the invasiveness of various cancer types. Yet, the dynamics of SSH1’s influence on liver cell stemness and circadian activity remain unclear. Through *in-silico*, tissue analysis, and functional assays, the study reveals a significant SSH1 expression in HCC samples, compared to non-cancerous counterparts, across six HCC platforms (AUC between 0.62 and 0.77, p < 0.01). The aberrant expression of SSH1 was correlated with poor patients’ survival (HR = 1.70, p = 0.0063) and progression-free (HR = 1.477, p = 0.0187) survival rates. Targeting SSH1, either via Sennoside A or CRISPR SSH1 in Huh7 cells (Huh7-SSH1-/-) significantly suppressed cell viability, migration, invasion, colony and tumorsphere formation of the Huh7-SSH1-/- cells. Mechanistically, we showed that downregulated SSH1 expression suppressed CLOCK, BMAL1, WNT3, β-catenin, LRP5/6, BCL2, VIM and Snail, with concomitant upregulated CFL-1/2, and CRY1 expression, indicating dysregulated circadian rhythm and WNT/β-catenin oncogenic pathway deactivation. Treatments in reflected notable tumor size reductions in the mice treated with SenAlight (1.76-fold, p < 0.01) and SenAdark (3.79-fold, p < 0.01). The expression of SSH1, CLOCK, BMAL1 and β-catenin proteins were significantly downregulated in the SenAlight and SenAdark mice; this was more so in the SenAdark mice. This reveals a potential treatment approach for HCC patients.

## INTRODUCTION

Liver cancer is the sixth most diagnosed malignancy and third most common cause of cancer-related mortality worldwide, with 905 677 new cases and 830 180 deaths in 2020 alone [1, 2]. Of the histological types of liver cancer, namely, cholangiocarcinoma (CC), focal nodular hyperplasia (FNH), hepatocellular adenoma (HCA), hepatocellular carcinoma (HCC), and combined hepatocellular carcinoma - cholangiocarcinoma (HCC-CC) [3], HCC accounts for about 75%, is mostly associated with chronic hepatic disease including liver cirrhosis, hepatitis B and hepatitis C [1, 2, 4]. Coupled with the rise in the annual number of new cases, patients with HCC are characterized by poor survival rates, such that the median survival time of inoperable cases ranges between 6 and 20 months, with a 5-year survival rate less than 5% [1, 2]. This may not be unconnected with patients presenting with advanced disease, vascular invasion, multifocal or multicentric large tumor, concomitant morbidities, or even organ failure [5].

Metastasis, a hallmark of cancer, remains a principal cause of cancer-related deaths, accounting for about 90% of all such deaths [6]. Epithelial-to-mesenchymal transition (EMT) and cancer stem cells (CSCs) are two major interrelated contributors to the metastasis of malignant cells [6, 7]. CSCs, originating from de-differentiated cells or tissue-resident stem cells, are a subset of malignant cells in the tumor bulk that inherently drive tumorigenesis, self-renewal of cancer cells, and cancer recurrence after initial treatment [6–8]. The accruing evidence indicating that cancer stemness is a reversibly acquired or lost cell state, and the phenotypic plasticity of CSCs, further accentuates the inter-relatedness of CSCs phenotypes and EMT, where the latter reflects the ability of cancer cells to switch cellular states [8, 9]. The activation of EMT is increasingly associated with the acquisition of CSCs-like traits, implicated in various cancer types, and induces resistance to contemporary chemotherapeutic agents [9–11]. EMT entails the loss of cellular apicobasal polarity, cytoskeletal rearrangement, and the acquisition of mesenchymal gene signature [9–12]. This transient loss of junctional complex proteins and acquisition of mesenchymal proteins facilitates the development of phenotypically more aggressive quasi-mesenchymal cell states in malignancies, including HCC [11–13]. The EMT-associated induction of actin structures and reorganization of cellular actin cytoskeleton facilitates enhanced cell migration and invasion [10–12]. The migration of malignant and non-tumor cells is a vital component of metastasis and depends on changes in the actin cytoskeleton typified by lamellipodia, filopodia, and invadopodia formation. These changes in the actin cytoskeleton are regulated by several actin-binding proteins such as cofilin-1 and slingshot protein phosphatase 1 (SSH1; also known as cofilin phosphatase or slingshot homolog 1) [14, 15].

The intricate interplay of biological processes in the human body is a testament to its complex regulatory mechanisms. At the heart of these mechanisms is the circadian rhythm, a natural, internal process that regulates the sleep-wake cycle and repeats on each rotation of the Earth roughly every 24 hours [16]. This rhythm does more than dictate our sleep patterns; it has an influential role in a multitude of physiological processes including hormonal secretion, cellular function, and metabolic regulation [16].

The intriguing intersection between SSHs and the circadian rhythm is an emerging area of research. Protein phosphatase 1 (PP1) is an example of a post-translational regulator of the mammalian circadian clock [17]. The slingshot family protein phosphatases (SSHs) currently compose of structurally overlapping but functionally distinct SSH1, SSH2 and SSH3 in mammals, and are reported to ‘specifically dephosphorylate and reactivate Ser-3-phosphorylated cofilin (P-cofilin)’, where upregulation of cofilin-1 has been shown to positively correlate with cancer aggression and metastasis [15]. SSH1 is activated by F-actin and the cofilin–phosphatase activity of SSH1 is strongly increased by F-actin binding, however, SSH1 exhibits two seemingly diametric activities in actin filament dynamics, namely, a cofilin–phosphatase activity that induces F-actin disassembly through cofilin-1 activation, and cofilin–phosphatase activity-independent stabilization and bundling of F-actin [18]. Despite this background understanding, if, how and to what extent SSH1 is involved tumor initiation, cancer metastasis, resistance to therapy, and poor prognosis remain largely under-explored, and more so compounded by our limited understanding of how the seemingly opposing actions of SSH1 are controlled in the context of the HCC microenvironment.

Hepatic homeostasis, a balance in the liver’s physiological processes, is essential for maintaining health. The liver, being a crucial metabolic organ, has many time-sensitive functions and, as such, is governed by the circadian rhythm [19]. This includes processes such as glucose metabolism, lipid processing, and detoxification pathways. Disruptions in this timing can lead to hepatic disorders and are often seen in conditions like fatty liver disease, cirrhosis, and liver enzyme abnormalities [19, 20]. Hepatocellular carcinoma (HCC), the primary form of liver cancer, is a culmination of numerous hepatic dysfunctions. The emerging body of research now suggests that circadian rhythm disturbances can influence the pathogenesis and progression of HCC [21]. There’s a growing understanding that aberrations in the circadian genes and proteins could play a role in liver tumorigenesis [22]. Additionally, factors that disrupt hepatic homeostasis, like metabolic disorders and inflammation, might act synergistically with circadian disruptions to enhance HCC risk. The existence of a temporal coordination of actin regulators has recently been suggested to drive cell-intrinsic actin dynamics, such that a presumed cellular clock controls and/or determines the efficiency of actin-dependent processes including cell adhesion and migration, thus ultimately impacting the efficacy of wound healing [23]. Since the ability of malignant cells to metastasize to other sites or organs depends on their ability to pervert and exploit natural wound-healing pathways [24], hypothesizing that SSH1 plays a vital regulatory role in the circadian modulation of the cellular actin cytoskeleton and that this influences cell motility, propensity to metastasize, treatment response, and ultimately the prognosis in patients with HCC.

The present demonstrated that SSH1 is significantly upregulated in HCC clinical samples, compared to their non-tumor counterpart and this aberrant expression of SSH1 is associated with worse overall and progression-free survival rates. We showed that pharmacological or CRISPR inhibition of SSH1 in HCC cell lines significantly suppressed cell viability, migration, invasion, colony and tumorsphere formation capabilities of the HCC cells. Mechanistically and of significant interest, we found that the molecular or pharmacological inhibition of SSH1 elicited suppression of CLOCK (circadian locomotor output cycles protein kaput), ARNTL/BMAL1 (aryl hydrocarbon receptor nuclear translocator like), WNT3 (Wnt family member 3), β-catenin, LRP5/6 (low-density lipoprotein receptor-related protein 5/6), BCL2 (B-cell CLL/lymphoma 2), vimentin (VIM) and Snail, with concomitant upregulated expression of cofilin-1/2 (CFL-1/2), and Cryptochrome (CRY), indicating dysregulated circadian rhythm and deactivation of the WNT/β-catenin oncogenic pathway. Consistent with these findings, we posit that the interplay between SSH1 and the ‘clock genes’ plays a critical role in actin cytoskeleton dynamics, the acquisition of cancer stemness phenotype, augmentation of cancer aggressiveness, HCC progression, and poor prognosis.

## RESULTS

### Slingshot protein phosphatase 1 (SSH1) is aberrantly expressed in hepatocellular carcinoma (HCC)

**Figure 1:**
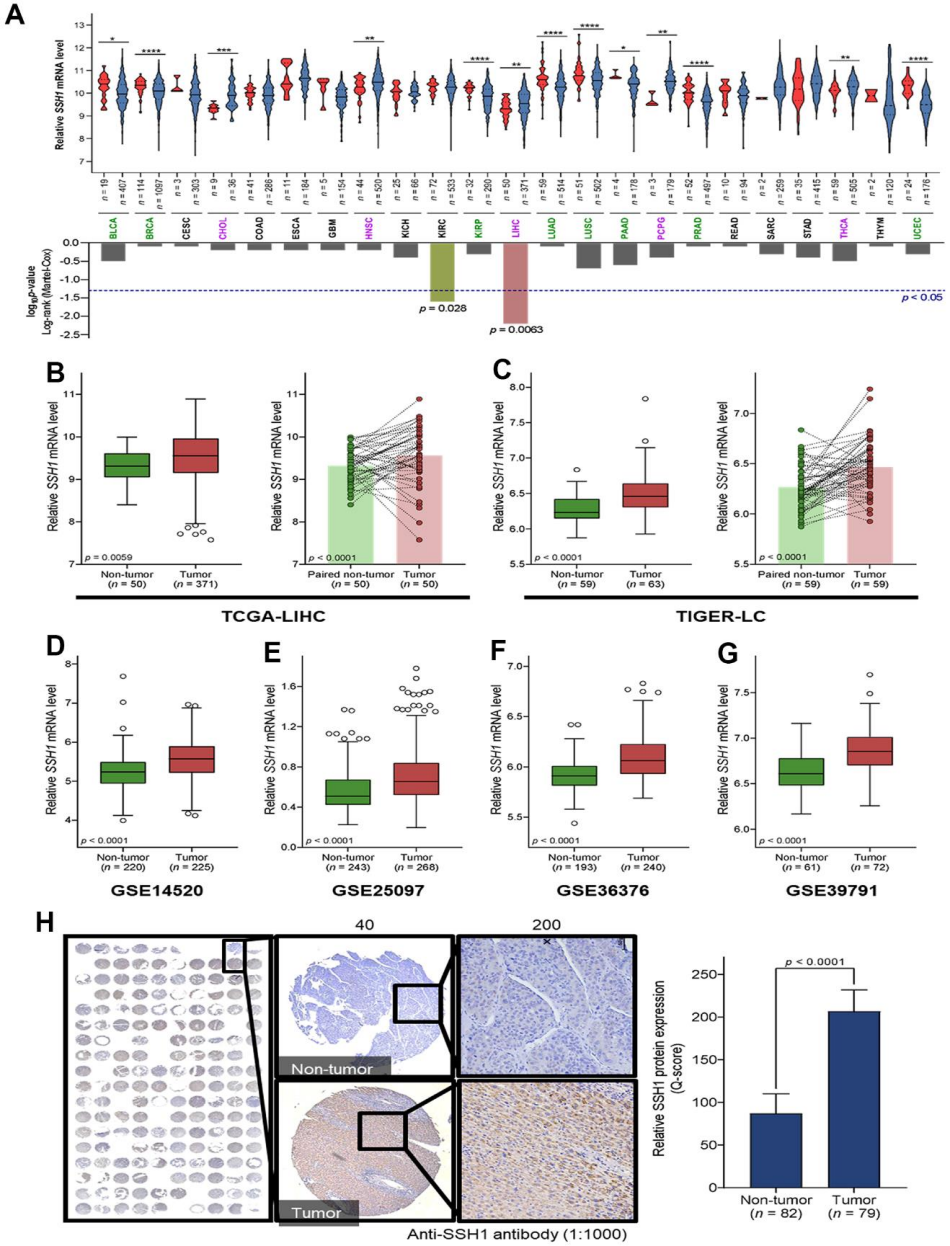
**SSH1 is aberrantly expressed in hepatocellular carcinoma (HCC).** (**A**) Violin plots with the graphical representation of the associated Mantel-Cox test showing the differential expression of SSH1 mRNA in 23 cancer types from the TCGA database. (**B**) Box plots of the relative SSH1 mRNA expression levels in the general non-tumor and tumor cases (*left*) or in paired non-tumor-tumor cases (*right*) of the TCGA-LIHC cohort. (**C**) Box plots of the relative SSH1 mRNA expression levels in the general non-tumor and tumor cases (*left*) or in paired non-tumor-tumor cases (*right*) of the TIGER-LC cohort. Box plots showing the relative SSH1 mRNA expression levels in non-tumor and tumor cases from the (**D**) GSE14520, (**E**) GSE25097, (**F**) GSE36376, and (**G**) GSE39791 HCC cohorts. (**H**) Representative IHC staining of HCC tissue microarray and histogram showing the protein expression of SHH1 in tumor and non-tumor based on the Q-score. *p < 0.05; **p < 0.01; ***p < 0.001.

Our analysis of the expression of SSH1 mRNA levels in tumor and non-tumor samples from patients with one of 23 cancer types contained in The Cancer Genome Atlas (TCGA) database showed that compared to the non-tumor samples, SSH1 was significantly upregulated in cholangiocarcinoma (CHOL) (*p* < 0.001), head and neck squamous cell carcinoma (HNSC) (*p* < 0.01), pheochromocytoma and paraganglioma (PCPG) (*p* < 0.01), thyroid cancer (THCA) (*p* < 0.01), and in liver hepatocellular carcinoma (LIHC, n = 421) (*p* < 0.01) (Figure 1A). More so, the Mantel-Cox test revealed significantly more total number of deaths among patients with higher expression of SSH1 than among their low expression group (*p* = 0.006) (Figure 1A). Minimizing likely experimental design-based bias, our analysis of paired tumor-non-tumor samples showed that compared to their paired non-tumor counterparts, SSH1 was overexpressed in the tumor samples from the TCGA-LIHC (n = 100; *p* < 0.0001) and the Thailand Initiative in Genomics and Expression Research for Liver Cancer (TIGER-LC) (n = 118; *p* < 0.0001) datasets (Figure 1B, 1C). Consistent with the above, we demonstrated similar mRNA expression profile for SHH1 in the GSE14520 (n = 445, *p* < 0.0001), GSE25097 (n = 511, *p* < 0.0001), GSE36376 (n = 433, *p* < 0.0001), and GSE39791 (n = 133, *p* < 0.0001) HCC datasets (Figure 1D–1G). Immunohistochemistry (IHC) of HCC tissue microarray constituting of paired samples from our HCC cohort confirmed the upregulation of SHH1 in tumor compared to non-tumor at protein level too based on the Q-score (2.36-fold, *p* < 0.0001) (Figure 1H).

### Overexpression of SSH1 is a biomarker of poor prognosis in patients with HCC

**Figure 2:**
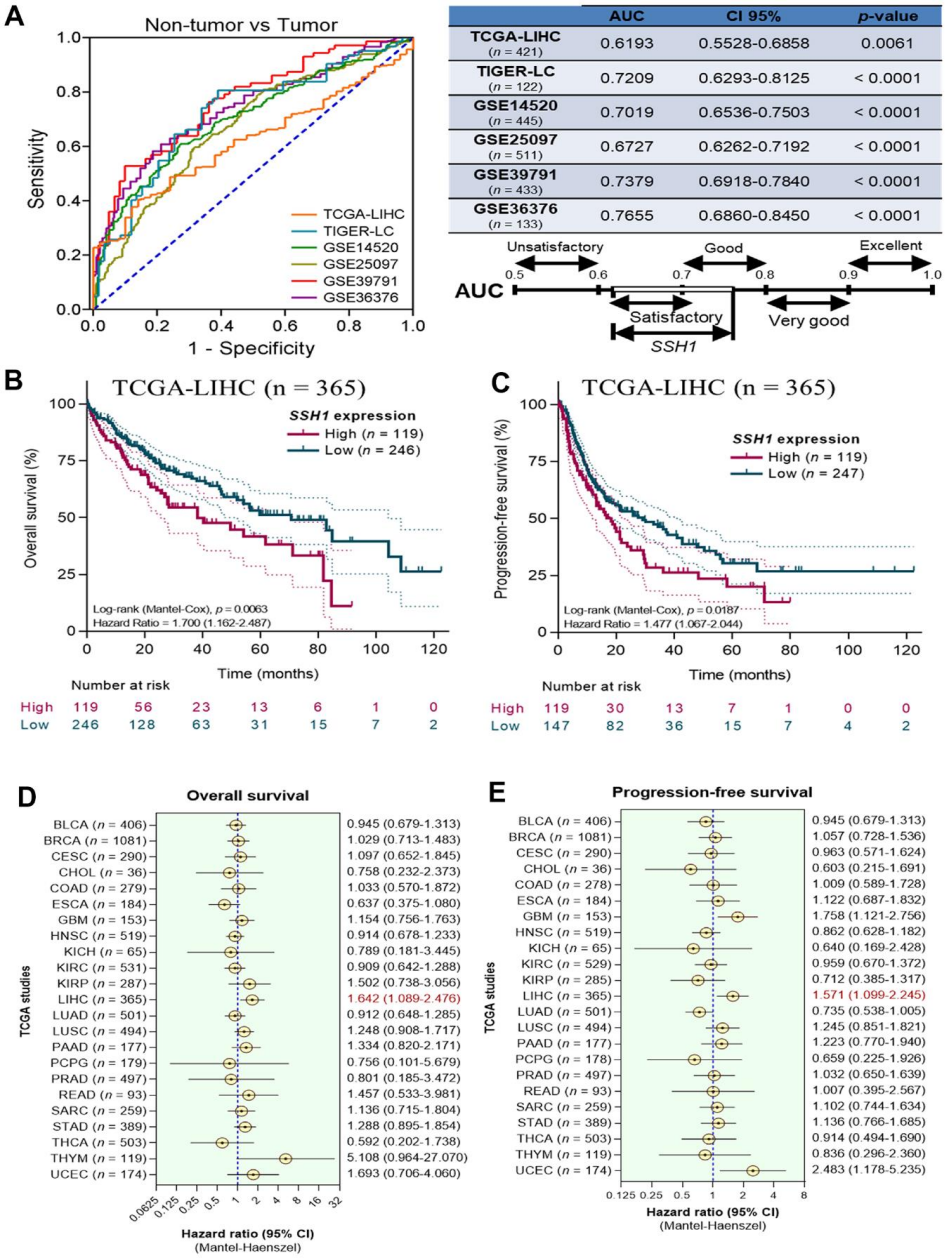
**Overexpression of SSH1 is a biomarker of poor prognosis in patients with HCC.** (**A**) Graphical representation (*left*) and statistical chart (*right*) showing the AUCs of TCGA-LIHC, TIGER-LC, GSE14520, GSE25097, GSE39791, GSE36376 of the non-tumor vs tumor samples. Kaplan-Meier plots of the (**B**) overall survival, and (**C**) progression-free survival in patients with high or low SSH1 expression from the TCGA-LIHC cohort. Forest plots of the (**D**) overall survival, and (**E**) progression-free survival based on SSH1 expression-based hazard ratios in patients with HCC from 23 different cancer types of the TCGA. All data are presented as means ± standard deviations of at least three independent experiments.

Furthermore, synchronous analysis of TCGA-LIHC (n = 421), TIGER-LC (n = 122), GSE14520 (n = 445), GSE25097 (n = 511), GSE39791 (n = 433), and GSE36376 (n = 133) HCC datasets demonstrated that the expression of SSH1 exhibits the satisfactory or good capability to differentiate non-tumor from tumor cases as expressed by the area under the receiver operating characteristics curves (AUCs) of 0.62 (*p* = 0.006), 0.72 (*p* < 0.0001), 0.70 (*p* < 0.0001), 0.67 (*p* < 0.0001), 0.74 (*p* < 0.0001), and 0.77 (*p* < 0.0001), respectively (Figure 2A). Having shown that SSH1 is significantly upregulated in HCC at mRNA and protein levels, with a suggested impact on survival, we next evaluated the effect of SSH1 differential expression on the survival of patients with HCC. Using the Kaplan-Meier survival plots, we found that compared to patients with low SSH1 expression, those with high SSH1 expression in the TCGA-LIHC cohort exhibited worse overall (OS; hazard ratio with 95% confidence interval, HR (95%CI) = 1.70, *p* = 0.006)) and progression-free (PFS; HR(95%CI) = 1.48, *p* = 0.02)) survival (Figure 2B, 2C). In addition, an analysis of OS and PFS across 23 cancer types from TCGA. Visualization of hazard ratios from the OS analyses using forest plots showed that second only to THYM (n = 119; hazard ratio, HR = 5.11), and UCEC (n = 174; HR = 1.69), patients with high expression of SHH1 were 1.6 times more likely to die of LIHC compared with their non-tumor counterparts (HR (95% confidence interval, CI) = 1.64 (1.09 - 2.48)) (Figure 2D). Similarly, our PFS analyses demonstrated that high SSH1 expression increased the likelihood of disease progression in patients with LIHC 1.6-fold (HR (95% CI) = 1.57 (1.1 - 2.24)), and this was second only to UCEC (HR (95% CI) = 2.48 (1.18 - 5.23)) and glioblastoma (GBM: HR (95% CI) = 1.76 (1.12 - 2.76)) (Figure 2E).

### SSH1 inhibition suppresses the viability, oncogenicity, and cancer stemness of HCC cells

**Figure 3:**
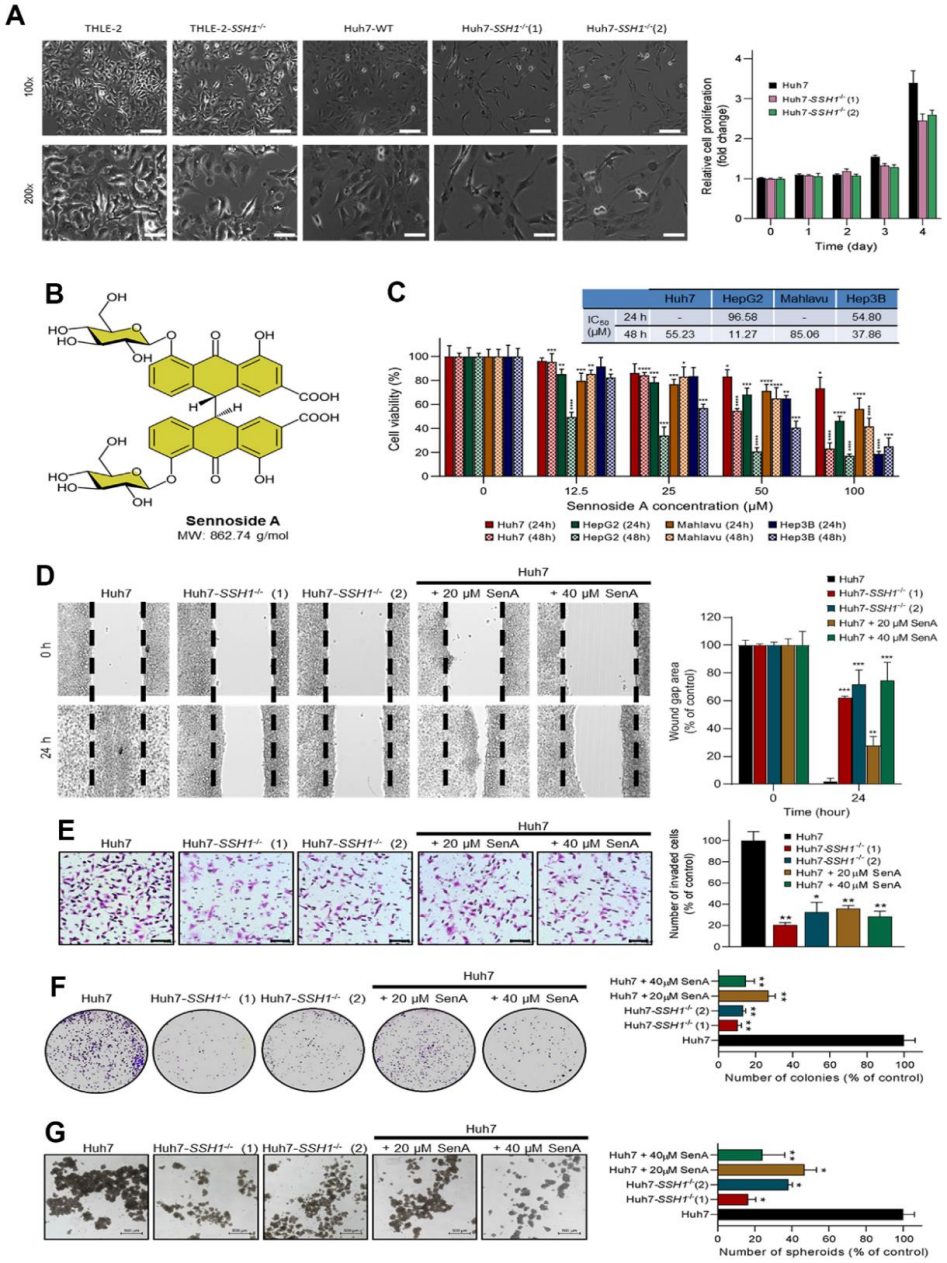
**SSH1 inhibition suppresses the viability, oncogenicity, and cancer stemness of HCC cells.** (**A**) Representative western blot images and histograms comparing cell viability in THLE-2, Huh7-WT, Huh7-SSH1^-/-^(1), and Huh7-SSH1^-/-^(2) cells at 100x and 200x magnification. (**B**) Molecular structure of Sennoside A with a molecular weight of 862.74 g/dl. (**C**) Histograms of the cell viability of Huh7, HepG2, Mahlavu, or Hep3B cells treated with 0 - 100 μM Sennoside A for 24h or 48h, with IC_50_ indicated. (**D**) Representative wound-healing migration images and histograms comparing the wound-gap closure in Huh7, Huh7-SSH1^-/-^(1), Huh7-SSH1^-/-^(2), 20 μM or 40 μM SenA-treated Huh7 cells. (**E**) Representative invasion assay images and histograms comparing the number of invaded Huh7, Huh7-SSH1^-/-^(1), Huh7-SSH1^-/-^(2), 20 μM or 40 μM SenA-treated Huh7 cells. (**F**) Representative colony-formation assay images and histograms comparing the number of colonies formed by Huh7, Huh7-SSH1^-/-^(1), Huh7-SSH1^-/-^(2), 20 μM or 40 μM SenA-treated Huh7 cells. (**G**) Representative tumorsphere-formation assay images and histograms comparing the number of tumorspheres formed by Huh7, Huh7-SSH1^-/-^(1), Huh7-SSH1^-/-^(2), 20 μM or 40 μM SenA-treated Huh7 cells. WT, wild type; SenA, Sennoside A; *p < 0.05; **p < 0.01; ***p < 0.001.

Furthermore, we used the Cox proportional hazard model for the assessment of our TMU-SHH HCC cohort clinicopathological data including gender, age, TMN stage, presence of metastasis, and SSH1 expression, as shown in Table 1. While the results of our univariate analysis showed that TMN stage (HR = 16.939, *p* = 0.0068), metastasis (HR = 9.971, *p* = 0.0030), and SSH1 expression Q-score (HR = 9.854, *p* = 0.0006) were strongly associated with HCC - related deaths, multivariate analysis revealed that only high SSH1 protein expression level is an independent predictor of disease-related death (HR = 7.272, *p* = 0.0042) (Table 1). The cut-off of 350 was ascribed for differential expression of SSH1 based on the Q-score derived from the intensity and distribution of SSH1 staining in the clinical samples. To better understand the oncogenic role of SSH1 in HCC, we performed a loss-of-function assay using the CRISPR/Cas9 technique and found that compared to the wild-type Huh7 (Huh7-WT) cells, cells with knocked-out SSH1 gene (Huh7-SSH1^-/-^ (1) and (2)) exhibited significantly reduced viability (Figure 3A, *left*) and proliferation over time, as expressed by a ~1.3-fold reduction in the proliferation of Huh7-SSH1^-/-^ cells (*p* < 0.05) (Figure 3A, *right*). Interestingly, knocking out SSH1 had no apparent effect on the viability or proliferation of normal human liver left lobe epithelial cell line THLE-2 cells (Figure 3A). Next, we demonstrated that the natural dianthrone glycoside called sennoside A (SenA), a popular herbal laxative for constipation with molecular weight 862.74 g/mol (Figure 3B) is a small molecule inhibitor of SSH1 and that exposure to 0 - 100 μM SenA dose-dependently suppressed the viability of Huh7 (IC_50_ = 55.23 μM), HepG2 (IC_50_ = 11.27 μM), Mahlavu (IC_50_ = 85.06 μM), or Hep3B (IC_50_ = 37.86 μM) HCC cell lines (Figure 3C). Interestingly, we found that similar to SSH1-/-, 20 μM and 40 μM Sen A significantly suppressed the migration of Huh7 cells, and this was dose-dependent (Figure 3D). Consistent with the above, we also demonstrated that cell invasion and colony formation were strongly inhibited in the Huh7-SSH1^-/-^ (1) and Huh7-SSH1^-/-^ (2) cells, and even more so in the Huh7 cells treated with 20 μM and 40 μM Sen A (Figure 3E, 3F). In addition, we demonstrated that compared with the wild type Huh7 cells, tumorsphere formation capability was significantly suppressed in the Huh7-SSH1^-/-^ (1) (5.56-fold, *p* < 0.05), Huh7-SSH1^-/-^ (2) (2.56-fold, *p* < 0.05), 20 μM (2.17-fold, *p* < 0.05), and 40 μM (3.85-fold, *p* < 0.01) Sen A - treated Huh7 cells (Figure 3G).

**Table 1:** Univariate and multivariate analyses of SSH1 expression in TMU-SHH HCC cohort.

**Clinicopathological variables**	**Patient, no.**	**Univariate analysis**			**Multivariate analysis**		
		**HR**	**95% CI**	**p-value**	**HR**	**95% CI**	**p-value**
**Gender** ** Male** ** Female**	10 49	0.464	0.060-3.593	0.4621	1.139	0.121-10.721	0.9097
**Age** **≤65** **>65**	30 29	1.009	0.325-3.129	0.9879	1.324	0.399-4.389	0.6464
**TMN** **Stage 1/2** **Stage 3/4**	32 27	16.939	2.184-131.364	0.0068*	0.200	0.015-2.588	0.2181
**Metastasis** **M0** **M1**	34 25	9.971	2.179-45.617	0.0030*	3.709	0.505-27.257	0.1977
**SSH1 expression** **Q-Score < 350** **Q-Score ≥ 350**	44 15	9.854	2.652-36.613	0.0006*	7.272	1.871-28.268	0.0042*

*, Statistically Significant.

### The oncogenic activity of SSH1 is mediated by circadian rhythm disruption and Wnt/β-catenin signaling activation, *in vitro*

**Figure 4:**
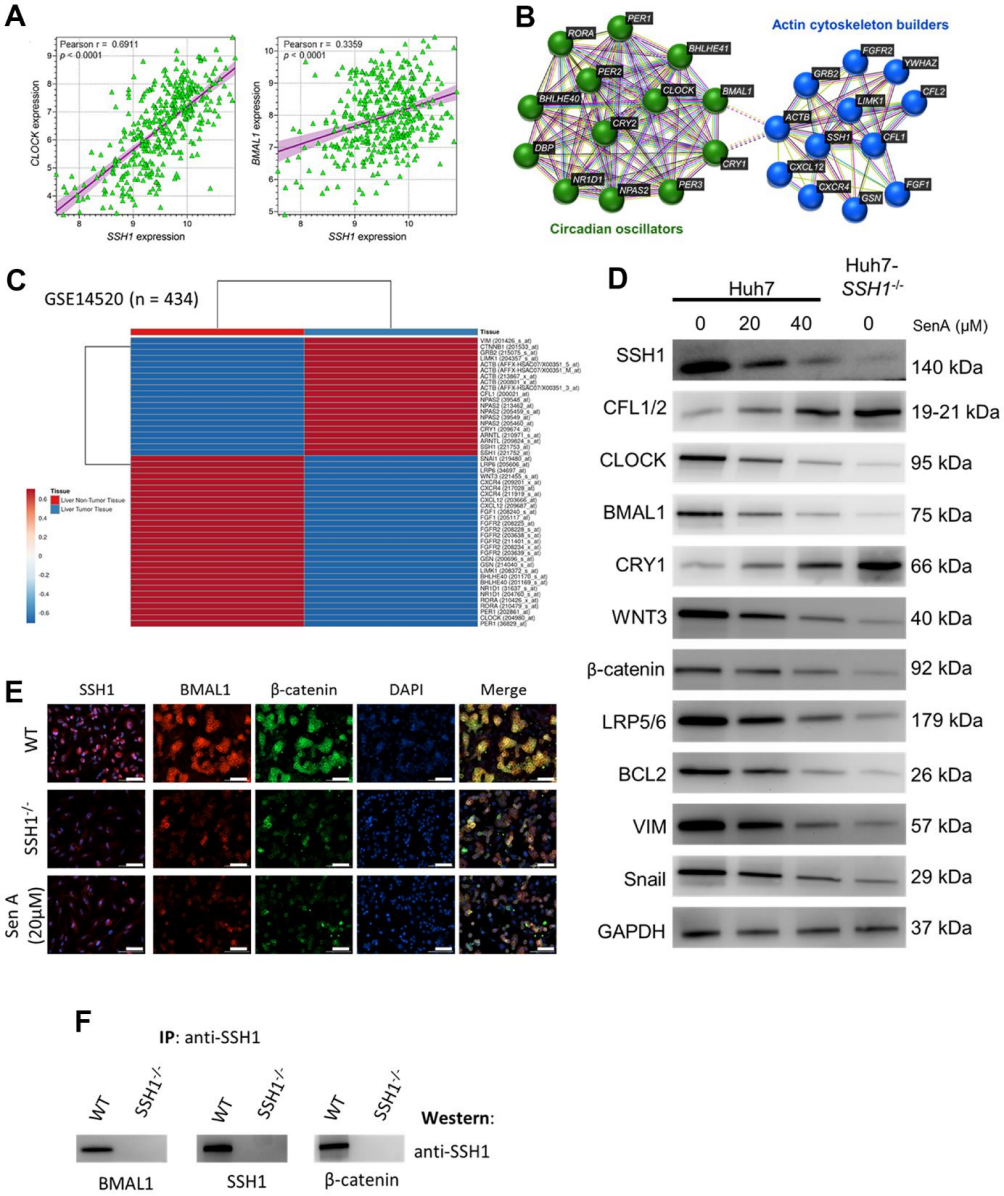
**The oncogenic activity of SSH1 is mediated by circadian rhythm disruption and Wnt/β-catenin signaling activation.** (**A**) Dot plots show the correlation between CLOCK and SSH1 expression (*left*), or BMAL1 and SSH1 expression (*right*). (**B**) The protein–protein interaction network constructed by the STRING database shows the interaction between SSH1, ‛actin cytoskeleton builders’, and ‛circadian oscillators’. (**C**) Heatmap of the expression profiles of VIM, CTNNB1, GRB2, LIMK1, ACTB, CFL1, NPAS2, CRY1, ARNTL, SSH1, SNAI1, LRP6, WNT3, CXCR4, CXCL12, FGF1, FGFR1, GSN, BHLHE40, NR1D1, RORA, PER1, and CLOCK. (**D**) Representative western blot images showing the effect of 0 - 20 μM SenA or Huh7-SSH1^-/-^ on the protein expression of SSH1, CFL1/2, CLOCK, BMAL1, CRY1, WNT3, β-catenin, LRP5/6, BCL2, VIM, and Snail. GAPDH was loading control. (**E**) Representative immunofluorescence staining images showing the expression and localization of SSH1, BMAL1, and β-catenin in Huh-WT, Huh7-SSH1^-/-^ and 20 μM SenA -treated Huh7 cells. DAPI stained for nuclear localization. (**F**) Representative IP western images showing the presence of BMAL1, SSH1, or β-catenin in IP complex of respective antisera on probing with SSH1 antisera in Huh-WT cells but not in the Huh-SSH1^-/-^ cells.

Against the background knowledge that cell-intrinsic actin dynamics is driven by the temporal coordination of actin regulators, and that this plays a critical role in the efficiency of actin-dependent processes such as the above-documented SSH1-related HCC aggression and progression, we probed for likely association between SSH1 expression and circadian rhythm modulators. We demonstrated a moderate to strong positive correlation between SSH1 and CLOCK (Pearson r = 0.69, *p* < 0.0001), or BMAL1 (Pearson r = 0.34, *p* < 0.0001) mRNA expression levels (Figure 4A). Using the STRING database (https://string-db.org) for visualization, we found that SSH1 interacts with actin cytoskeleton proteins, including CFL1, CFL2, Actin Beta (ACTB), Actin-Depolymerizing Factor/Gelsolin (GSN), LIM Domain Kinase 1 (LIMK1), Growth Factor Receptor Bound Protein 2 (GRB2), and forms a cascade with circadian oscillators, namely CLOCK, BMAL1, CRY1, CRY2, Period Circadian Regulator (PER)1/2/3, RAR Related Orphan Receptor A (RORA), and Basic Helix-Loop-Helix Family Member (BHLHE)40/41 (Figure 4B). In concordance, analysis of the GSE14520 HCC dataset (n = 434) found that while the median expression of SSH1, ARNTL/BMAL1, ACTB, LIMK1, GRB2, CTNNB1/β-catenin, and VIM were upregulated, CXCR4, CXCL12, GSN, BHLHE40, BHLHE41 and RORA were downregulated in liver tumor tissues (Figure 4C). Furthermore, we demonstrated that similar to the protein expression profiles elicited in the Huh7-SSH1^-/-^ cells, treatment with 20 μM and 40 μM Sen A elicited significant dose-dependent suppression of SSH1, CLOCK, BMAL1, WNT3, β-catenin, LRP5/6, BCL2, VIM and Snail, with concomitant upregulation of CFL1/2, and CRY1 proteins expression levels (Figure 4D). We also showed the colocalization and significantly co-reduced protein expression of cytomembrane SSH1, BMAL1, and β-catenin in the Huh7-SSH1^-/-^ cells, and upon exposure to 20 μM SenA (Figure 4E). In addition, based on co-immunoprecipitation, we found that more than colocalization and coexpression, SSH1 directly binds with BMAL1 and β-catenin (Figure 4F). These data do indicate that the oncogenic activity of SSH1 is mediated by circadian rhythm disruption and Wnt/β-catenin signaling activation.

### The oncogenic activity of SSH1 is mediated by circadian rhythm disruption, *in vivo*

**Figure 5:**
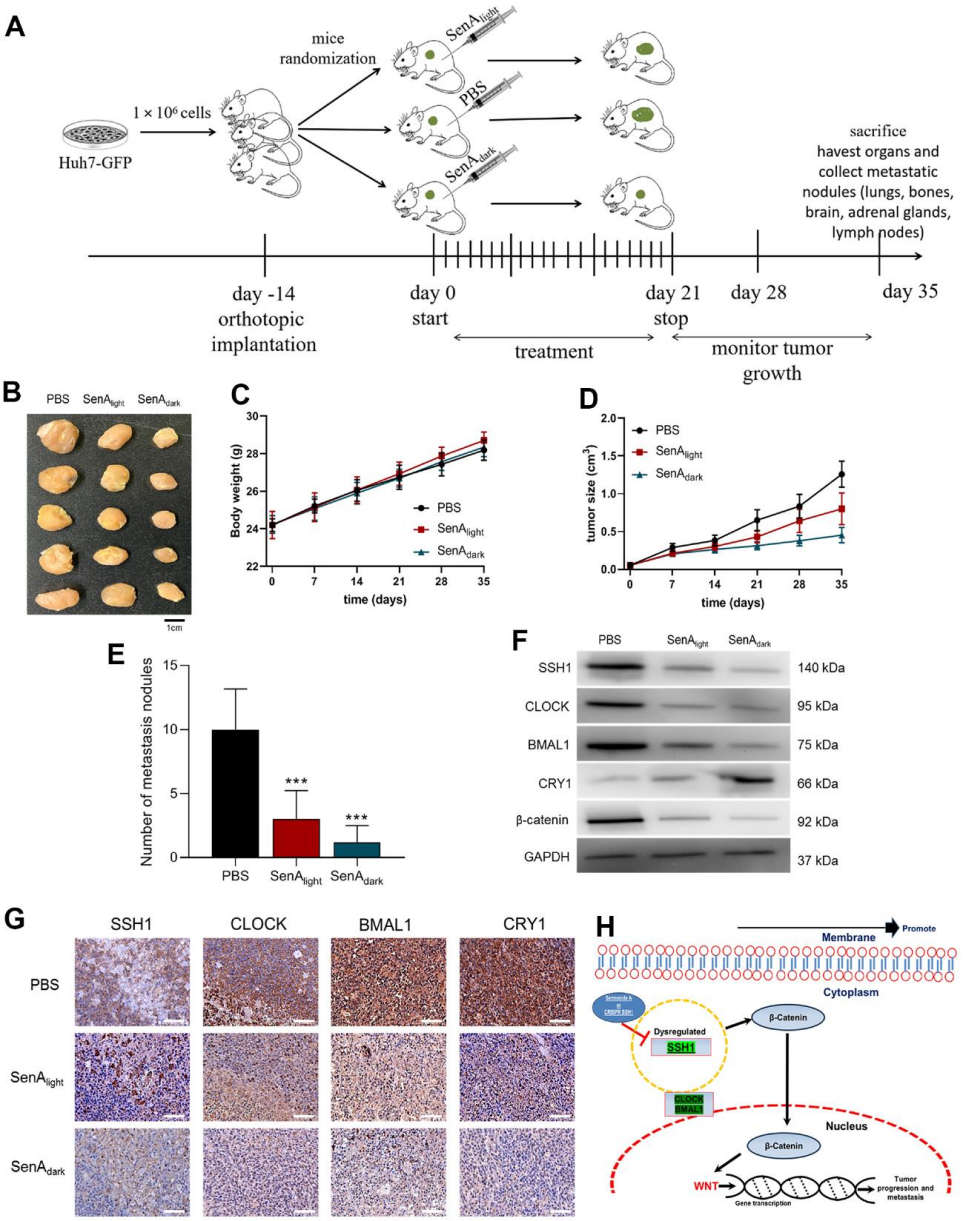
The oncogenic activity of SSH1 is mediated by circadian rhythm disruption, *in vivo* (**A**) Workflow schema of the orthotopic HCC mice model generation. (**B**) Photo-image showing the differential effect of SenAlight and SenAdark on the tumor sizes in xenografted mice. Line graphs showing the differential effects of SenAlight and SenAdark on the (**C**) average tumor volume in and (**D**) body weight of the treated mice compared with the PBS control mice, at the indicated time-points. (**E**) Histograms showing how PBS, SenAlight and SenAdark affect the number of intrahepatic and lung metastasis nodules. (**F**) Representative western blot images of the effect of PBS, SenAlight and SenAdark on the expression levels of SSH1, CLOCK, BMAL1, CRY1, and β-catenin in dissociated HCC cells from tumors extracted from xenografted mice. GAPDH served as a loading control. (**G**) Representative tissue staining images showing the effect of PBS, SenAlight and SenAdark on the staining intensity and distribution of SSH1, CLOCK, BMAL1, and CRY1 proteins in tumors extracted from xenografted mice. (**H**) a pictorial description of the mechanism of regulation of HCC progression via SSH1-β-catenin and WNT signaling. The data are presented as the mean ± standard error of mean (SEM), n = 5/group.

Having demonstrated that SSH1 expression and/or activity is associated with the disruption of circadian rhythm and activation of Wnt/β-catenin signaling *in vitro*, we next sought to see if this is replicated *in vivo* using NOD/SCID mice inoculated with 1 x 10^6^ GFP-labeled Huh7 cells (Figure 5A). As shown by our *in vivo* data, compared with the PBS-treated control mice (PBS, n = 5), mice treated with 10 mg/kg/day SenA intraperitoneally at 9 am (SenA_light_), or at 9 pm (SenA_dark_) for 21 days elicited dramatically reduction in tumor burden, respectively by day 35 (Figure 5B). Similarly, significantly reduced tumor volume was observed in the mice treated with SenA_light_ (1.76-fold, *p* < 0.01) and SenA_dark_ (3.79-fold, *p* < 0.01) (Figure 5C). No apparent difference was observed between the body weights of mice from the PBS control, SenA_light_, or SenA_dark_ group (Figure 5D). Furthermore, we found that the SenA_dark_ mice exhibited the least intrahepatic and lung metastasis, compared with the SenA_light_ and PBS-treated groups (Figure 5E). Also, using cells dissociated from tumors extracted from the mice, in comparison to the PBS-treated group, the expression of SSH1, CLOCK, BMAL1 and β-catenin proteins were significantly downregulated in the SenA_light_ and SenA_dark_ mice; this was more so in the SenA_dark_ mice (Figure 5F). Conversely, compared with the PBS control and SenA_light_ mice, CRY1 protein expression was upregulated in the SenA_dark_ mice (Figure 5F). Consistent with the western blot results, we demonstrated that the circadian nature of the tumors in PBS, SenA_light_ and SenA_dark_ mice maintained under specified light/dark conditions, with SSH1-mediated reduced immunoreactivity of SSH1, CLOCK, BMAL1, and CRY1 in tumors from the SenA_light_ and SenA_dark_ (Figure 5G). These data indicate that the oncogenic activity of SSH1 has a circadian rhythm molecular underlining and that this can be exploited for therapeutic benefit of patients with HCC (depicted in Figure 5H).

## DISCUSSION

The formation and progression of HCC is characterized by changes in the gene expression profile of hepatocytes, and this consequently leads to the acquisition of malignant, aggressive, and often therapy-resistant phenotypes observed in cancerous hepatic cells [3–6, 10, 25]. Several studies have suggested that SSH1 may play some critical roles in the maintenance of protein structure and stability, regulation of cellular localization, function and activities, as well as the homo- and hetero-dimeric protein-to-protein interactions, with an apparent dearth of information on its complicity in HCC [14]. Over the last decade, there has been reports suggesting that cell migration, an essential component of metastasis typified by altered actin cytoskeleton such as lamellipodia, filopodia, and invadopodia formation (regardless of their cancerous status), is regulated by certain actin-binding proteins including the cofilin phosphatase SSH1 [15].

Recently, there have been reports indicating that upregulated SSH1 expression was significantly associated with metastasis and strongly correlated with poor prognosis in patients with gastric and colorectal cancers [26]. Consistent with these reports, the present study demonstrated, for the first time to the best of our knowledge, that SSH1 is aberrantly expressed in HCC and that this overexpression of SSH1 is prognostic of poor clinical outcome in patients with HCC (Figures 1, 2). The clinical significance and statistical relevance are evident when the Kaplan-Meier plots indicate that patients exhibiting high SSH1 levels face a heightened risk of mortality and disease progression. The utility of receiver operating characteristics (ROC) curves in disease diagnosis and prognosis cannot be overstated. Their application is especially pertinent when discerning the discriminative ability of biomarkers like SSH1 or when validating its role as a diagnostic and prognostic indicator. In the realm of biomarker identification and validation, the Area Under the Curve (AUC) is a renowned measure of the overall accuracy of discrimination. A higher AUC value signifies the superior discriminatory prowess of a given diagnostic or prognostic tool [27]. Within this study, the observed AUC ranged from 0.62 to 0.77, which is satisfactory, underscoring the importance of SSH1 as a pivotal biomarker. This observation is further corroborated by subsequent experiments.

Our data showing that the molecular inhibition or pharmacological targeting of SSH1 suppressed the viability, oncogenicity, and cancer stemness of HCC cells (Figure 3), is also of clinical relevance, and in line with similar findings in colorectal cancer, wherein the shRNA-mediated loss of SSH1 significantly impaired the migration, invasion, and colony formation of colorectal cells, *in vitro* [28]. Cancer stem cells (CSCs) are intrinsically self-renewing, and as such their presence or enrichment confer the so-called cancer stemness phenotypes, which are increasingly implicated in resistance to metastasis, chemotherapy, and cancer recurrence, which continue to constitute a major clinical challenge to effective anticancer treatment, especially as an effective CSC eradication strategy remains elusive [6–11, 29]. It is thus noteworthy that targeting SSH1 strongly suppressed cancer stemness phenotypes, including colony and tumorsphere formation by HCC cells, and may be exploited for the development of a CSCs elimination-based anticancer therapeutic strategy for patients with HCC.

It is also of therapeutic relevance that we found that the oncogenic activity of SSH1 is mediated by circadian rhythm disruption and Wnt/β-catenin signaling activation (Figure 4). Consistent with these findings, significantly reduced tumor volume was observed in the mice treated with Sennoside A (p < 0.01). The expression of SSH1, CLOCK, BMAL1 and β-catenin proteins were significantly downregulated in the Sennoside A_dark_ treat mice (Figure 5), indicating that the oncogenic activity of SSH1 has a circadian rhythm molecular underlining and that this can be exploited for the therapeutic benefit of patients with HCC. There is evidence that aberrant Wnt/β-catenin signaling facilitates the renewal of CSCs, proliferation and differentiation of cancer cells, thus playing a vital role in tumor formation, cancer progression, and therapy resistance [30]. This vital role informs continued research efforts to demystify this Wnt/β-catenin signaling, identify its activators, and in so doing, develop specific and targeted antagonists or inhibitors of this oncogenic pathway as effective therapeutic strategies for personalized anticancer treatment. Our findings indicating that the oncogenic activity of SSH1 is mediated by circadian rhythm disruption and Wnt/β-catenin signaling activation is consistent with recent reports that low levels of the period circadian regulator 3 (PER3) induces the BMAL1 expression, leads to β-catenin phosphorylation and activation of the WNT/β-catenin signaling pathway, and is characteristic of ALDH^hi^CD44^+^ prostate CSCs [31]. This further emphasizes the clinical benefits of targeting SSH1 in malignant diseases, including HCC.

To reiterate, the circadian rhythm, often referred to as the circadian clock, adjusts, shapes, and synchronizes external stimuli and environmental shifts with the organism’s internal processes [23, 32]. In essence, this clock ensures the organism’s timely accuracy and strong adaptability to its environment. Disruptions or misalignments in the circadian rhythm can elevate the risk of systemic illnesses, including cancer [32]. Our current research indicates that when SSH1 expression is decreased, there is a suppression in the ‛clock proteins’ CLOCK, BMAL1, and metastasis markers like WNT3, β-catenin, LRP5/6, BCL2, VIM, and Snail. Concurrently, there’s an increase in ‛clock proteins’ CFL-1/2, and CRY1. These findings enrich our understanding of how an imbalanced circadian rhythm can fuel cancer-causing signals. They also underscore the potential of targeting circadian signals as a promising clock-based treatment approach for HCC patients. These insights bolster the emerging field of circadian medicine. Within this domain, therapeutic methods, such as SSH-/- or Sennoside A, which focus on central clock components and their immediate regulators to modify the circadian rhythm, are being refined to maximize their therapeutic efficacy [32]. Moreover, the impact of SSH1 can be incorporated into HCC treatment strategies through circadian rhythm modulation. Nonetheless, additional research is required to reaffirm and establish a significant correlation between SSH1 and downstream signaling in HCC.

**Figure 6:**
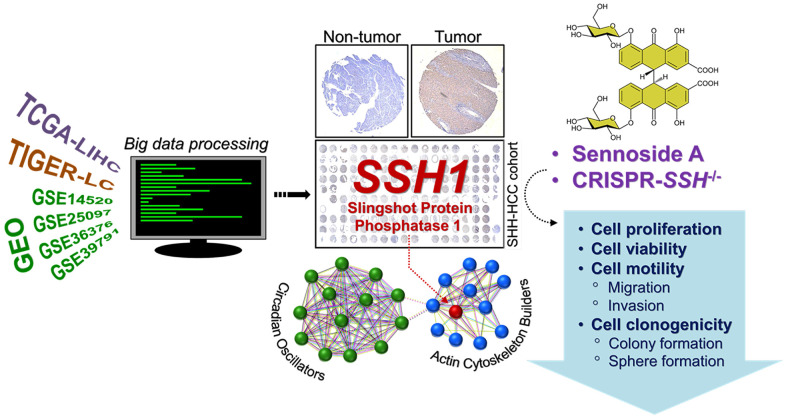
Graphical abstract showing that the oncogenic activity of SSH1 is mediated by circadian rhythm disruption and Wnt/β-catenin signaling activation, and that SHH1 is molecular and pharmacological target in patients with HCC.

In conclusion, as depicted in Figure 6, our study highlights that SSH1 serves as an actionable molecular biomarker associated with actin-binding, stemness, and circadian rhythm modulation. Its therapeutic and prognostic potential for HCC patients is notably significant.

## MATERIALS AND METHODS

### Drug and reagents

Sigma Aldrich® Sennoside A (#01870575) obtained from Merck (Merck KGaA, Darmstadt, Germany) was dissolved in dimethyl sulfoxide (DMSO; #D2650; Merck KGaA, Darmstadt, Germany) to generate 10mM stock solutions and stored at −20° C. Phosphate buffered saline (PBS, #P7059), trypsin/ethylenediaminetetraacetic acid (Trypsin-EDTA, #T4049) solution, sulforhodamine B (SRB) reagent (#230162), acetic acid (#695092), and trisaminomethane (Tris) base (#93352) were purchased from Sigma Aldrich Co. (St. Louis, MO, USA). Primary antibodies against SSH1 (#13578), β-catenin (#8480), BCL2 (#15071), VIM (#46173), Snail (#3879) were purchased from Cell Signaling Technology (Boston, MA, USA), LRP5/6 (#ab36121), CLOCK (#ab178525), BMAL1 (#ab235577), CRY1 (#ab171860), WNT3 (#ab116222) from Abcam (Abcam plc., Cambridge, UK), CFL1/2 (#sc-376476; Santa Cruz Biotechnology, Santa Cruz, CA, USA), and GAPDH (#60004-1-Ig; Proteintech Group Inc., Rosemont, IL, USA). The membranes were incubated in primary antibodies in Supplementary Table 1.

### Cell lines and culture

The human hepatocellular carcinoma Huh7 (JCRB0403) from the NIBIOHN (National Institute of Biomedical Innovation, Health and Nutrition, Japanese Collection of Research Bioresources (JCRB) Cell Bank, Japan), Mahlavu, HepG2 (HB-8065™) and Hep3B (HB-8064™) HCC cells, as well as the normal human liver left lobe epithelial cell line THLE-2 (CRL-2706™), were purchased from the American Type Culture Collection (ATCC, Manassas, VA, USA). The cells were cultured in DMEM (Gibco-Life Technologies Inc., Gaithersburg, MD, USA) or RPMI1640 (Invitrogen, San Diego, CA, USA) supplemented with 10% fetal bovine serum (FBS, Invitrogen), 100 μg/mL streptomycin and 100 UI/mL and penicillin at 37° C in humidified 5% CO2 atmosphere. Cells were passaged at ≥ 90% confluence or culture media changed every 72 h.

### SSH1 knockout by CRISPR/Cas9

The Huh7 cell line with *SSH1* gene knockout (Huh7-*SSH1*^-/-^) were generated using the CRISPR/Cas9 technique. Two sgRNAs targeting the 4th and 5th coding exon of the *SSH1* gene were separately cloned into pAll-Cas9-pPpuro plasmid from CRISPR Gene Targeting Core Lab (TMU Research Center of Cancer Translational Medicine, Taipei Medical University, Taipei, Taiwan). The following targeting sites were used: 5’-TTCTGTCTTCGCAACGCAGAAGG-3’ for sgRNA#1 and 5’-GGACCGGGTCCGG TACATGGTGG-3’ for sgRNA#2. Two sgRNA plasmids (2 μg) were transfected into Huh7 cells by Lipofectamine 3000 transfection reagent. On day 2 post-transfection, the transfected cells were selected with 2 μg/mL puromycin for one week. Viable single cell clones were isolated by limiting dilution into a 96-well plate. The Huh7-*SSH1*^-/-^ cells were double-confirmed by Western blot analysis and DNA sequencing of the genomic regions.

### HCC data acquisition

Raw mRNA-sequencing data with clinical parameters for HCC cohorts were acquired from the freely accessible online cancer databases, namely the Gene Expression Omnibus (GEO) (https://www.ncbi.nlm.nih.gov/geo/), Genotype-Tissue Expression (GTEx) (https://www.gtexportal.org), The Cancer Genome Atlas (TCGA) (https://xenabrowser.net/), and R2: Genomics Analysis and Visualization Platform. Furthermore, all data analyses were performed in the R programming environment (version 3.2.5) and the graph was created using GraphPad Prism 5.0 software (GraphPad Software, Inc., La Jolla, CA, USA).

### HCC specimens and cohort characterization

HCC clinical samples retrieved from the Taipei Medical University — Shuang Ho Hospital (TMU—SHH) HCC tissue archive, were used in the present study.

### Immunohistochemistry

Immunohistochemical (IHC) staining and quantitation were performed according to standard protocol. Briefly, we blocked endogenous peroxidase activity using 3% hydrogen peroxide after xylene-based de-waxing and ethanol re-hydration of the 5μm thick sections. Thereafter, antigen was then retrieved and blocked with 10% normal serum, before incubating the sections with anti-SSH1 antibody (1:1000; HPA019845, Sigma Aldrich) at 4° C overnight, and goat anti-rabbit IgG horseradish peroxidase (HRP)-labeled secondary antibody (1:10,000; #65-6120, Thermo Fisher Scientific Inc., Waltham, MA, USA). Diaminobenzidine (DAB) was used as chromogenic substrate, and stained tissue sections were counter-stained with hematoxylin (Thermo Fisher Scientific, Waltham, MA, USA). Univariate and multivariate analyses were carried out using the Cox proportional hazards regression model.

### Sulforhodamine B assay

Huh7 (8×10^3^), Mahlavu (8×10^3^), HepG2 (6×10^3^) or Hep3B (4×10^3^) HCC cells were seeded per well onto 96-well plates. After 24 hours of cultivation, the cells were treated with 0 - 100 μM sennoside A prepared in DMSO. Cell viability was assessed at 24 and 48 hours after treatment in the Spectramax M3 multimode microplate reader (Molecular Devices LLC., San Jose, CA, USA) at a wavelength of 570 nm.

### Scratch-wound migration assay

Wild type (Huh7-*SSH1* WT) and Huh7-*SSH1*^-/-^ cells were seeded (5×10^4^ cells/chamber) onto 2 well silicone culture inserts (ibidi GmbH, Gräfelfing, Germany) placed in the wells of 24-well plates containing DMEM supplemented with 0.2% FBS. After the cells were cultured to ≥ 95% confluence, the inserts were removed, the attached cell areas were carefully washed with cold PBS two times, and then fresh medium with or without 20-40 μM sennoside A added. The scratch-wound areas were captured under a microscope at 0 and 24 hours after treatment. Consequently, quantification was performed with ImageJ software (https://imagej.nih.gov/ij/).

### Transwell matrigel invasion assay

Millicell culture inserts (Merck KGaA, Darmstadt, Germany) were pre-coated with BD matrigel matrix (Sigma-Aldrich) at 4° C overnight. The Huh7-*SSH1* WT and Huh7-*SSH1*^-/-^ cells were then seeded onto the membrane (1×10^5^ cells/chamber) in DMEM supplemented with 1% FBS with or without 20-40 μM sennoside A. The lower chambers contained DMEM with 10% FBS. After 18-hour incubation, cells that did not invade remaining on the top surface of membranes were gently discarded using a sterile cotton swab, while the invaded cells were fixed in methanol, followed by crystal violet staining, imaging and consequent quantification under the microscope.

### Colony formation assay

Four hundred Huh7-*SSH1* WT and Huh7-*SSH1*^-/-^ cells were seeded per well onto 6-well plates containing DMEM supplemented with 10% FBS with or without 20-40 μM sennoside A. After two weeks of culturing in 5% humidified CO_2_ incubator at 37° C, formed colonies were fixed in 4% formaldehyde and stained using 0.1% crystal violet in methanol. The former colonies were then photographed and counted.

### Tumorsphere formation assay

HCC cells (5 × 10^3^/well) were seeded into ultra-low-attachment 24-well plates containing serum-free medium supplemented with 10 ng/mL human basic fibroblast growth factor (Invitrogen, Carlsbad, CA, USA), 1x B27 supplement (Invitrogen), and 20 ng/mL epidermal growth factor (Invitrogen). The medium was replaced every 72 hours. After two weeks, formed tumorspheres were photographed and counted under a microscope.

### *In vivo* circadian rhythm experiments

All animal experiments were approved by the institutional animal care and use committee of Taipei Medical University (LAC2022-0488). NOD/SCID mice (male, 6-8 weeks; mean weight: 23 ± 2.1 g) purchased from BioLASCO Taiwan Co., Ltd. (Taipei City, Taiwan) were used in this study. The mice were housed under a 12h: 12h Light: Dark condition and allowed access to food and water *ad libitum* for a week before tumor implantation for acclimatization. HCC Huh7 cell lines were cultured as already described in RPMI-1640 medium supplemented with 10% FBS (Gibco, Waltham, MA, USA). The Huh7 cells were transfected with pLVXEGFP1-C1 (Cat#.632155. Clontech Lab.Inc., Takara Bio., Mountain View, CA, USA) following the manufacturer’s instructions. Huh7-GFP cells (2 × 10^6^) were re-suspended in 25 μL of PBS/Matrigel (1:1) and inoculated under the capsule of the left hepatic lobe of the mice. 14 days after tumor cell inoculation (Day 0), the mice now bearing apparent orthotopic tumor xenografts were randomized into 3 experimental groups, namely control (PBS, n = 5), SenA_light_ (n = 5; 10 mg/kg/day SenA, i.p., 9 am), and SenA_dark_ (n = 5; 10 mg/kg/day SenA, i.p., 9 pm). SenA (i.p., once daily) was given for 21 days. Tumor formation and metastatic progression were monitored.

### Statistical analysis

All results represent the mean ± standard deviation (SD) of assays performed at least three times in triplicates. The two-sided Student’s t-test was used to compare two groups, while one-way ANOVA with a Tukey post hoc test was used for comparisons between multiple pairs of groups. Forest plots were generated for visualization of hazard ratios from the overall and progression-free survival analyses of various TCGA datasets. All data were visualized and analyzed using SPSS Statistics 21.0 (IBM Corporation, NY, USA), and the GraphPad Prism version 9.0 for Windows (GraphPad Software, Inc., La Jolla, CA, USA). Protein-protein interaction network was constructed on STRING: functional protein association networks (https://www.string-db.org). P-value < 0.05 was considered statistically significant.

### Availability of data and materials

The datasets used and analyzed in the current study are publicly accessible as indicated in the manuscript. Experimental procedures, characterization of new compounds, and all other data supporting the findings are available in the Supplementary Materials. Supplementary Figure 1. Full-size blots of Figure 4D, 4F and Supplementary Figure 2. Full-size blots of Figure 5F

## Supplementary Material

Supplementary Figures

Supplementary Table 1
