# Supplementary material for: Dysregulated expression of slingshot protein phosphatase 1 (SSH1) disrupts circadian rhythm and WNT signaling associated to hepatocellular carcinoma pathogenesis
Source: Aging (Albany NY). 2023 Oct 13;15(20):11033–51. doi: 10.18632/aging.205064 (PMC10637823; doi:10.18632/aging.205064)
Supplement: Supplementary Figures [file aging-15-205064-s001.pdf]

SUPPLEMENTARY FIGURES

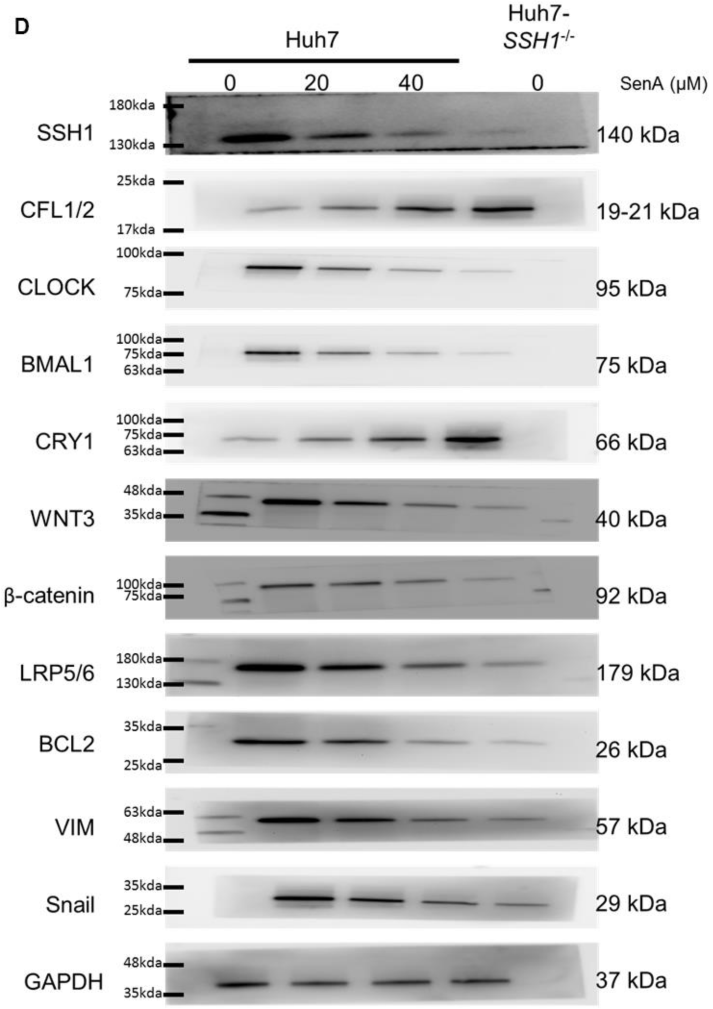

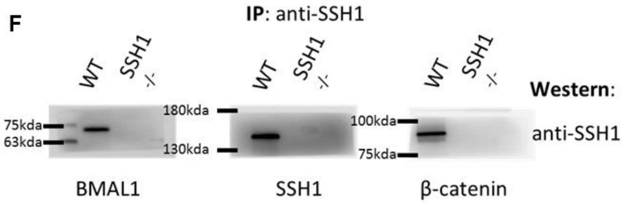

Supplementary Figure 1. Full-size blots of Figure 4D and 4F.

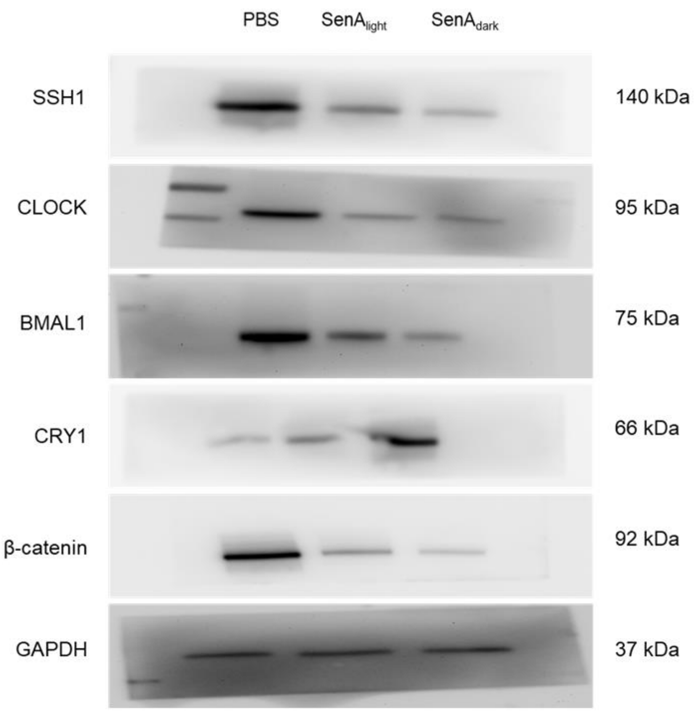

Supplementary Figure 2. Full-size blots of Figure 5F.
