# Supplementary material for: Dysregulated expression of slingshot protein phosphatase 1 (SSH1) disrupts circadian rhythm and WNT signaling associated to hepatocellular carcinoma pathogenesis
Source: Aging (Albany NY). 2023 Oct 13;15(20):11033–51. doi: 10.18632/aging.205064 (PMC10637823; doi:10.18632/aging.205064)
Supplement: Supplementary Table 1 [file aging-15-205064-s002.pdf]

SUPPLEMENTARY TABLES

Supplementary Table 1. The membranes were incubated in primary antibodies.

No.	Target	Dilution	MW (kDa)	Source	
1	LRP5/6	1:1000	179	ab36121	Abcam
2	CLOCK	1:1000	95	ab178525	Abcam
3	BMAL1	1:1000	75	ab235577	Abcam
4	CRY1	1:1000	66	ab171860	Abcam
5	WNT3	1:1000	40	ab116222	Abcam
6	β-catenin	1:1000	95	#8480	Cell signaling
7	SSH1	1:1000	140	#13578	Cell signaling
8	BCL2	1:1000	26	#15071	Cell signaling
9	VIM	1:1000	57	#46173	Cell signaling
10	Snail	1:1000	29	#3879	Cell signaling
11	CFL1/2	1:1000	19-21	sc-376476	Santa Cruz
12	GAPDH	1:10000	37	60004-1-Ig	Proteintech
